# The Oracles of Medicine

**Published:** 1887-10-01

**Authors:** 


The Hospital

A Weekly Institutional Journal of

Science, Medicine, and Philanthropy.

*For Hospitals, Asylums, Medical Practitioners, Students, Nurses, Families, Parishes, Congregations, and the Charitable Public.*

Vol. III. No. 53.] [October 1, 1887.

### The Oracles of Medicine.

It used to be said that "every man was a fool or a physician at forty." The saying arose when a physician was a very different person from what he is now, and when every man was considered a fool, or rather an unlearned person, who did not possess a more or less considerable knowledge of everything. Aristotle was the parent of innumerable philosophical descendants who took "all knowledge" for their province; and if we consider that they lived no longer than the allotted fourscore years, we cannot but be astonished at the very creditable show many of them made in many subjects. It was reserved for the men of recent times to be less broad, but more profound and accurate. The modern scientific man is truly admirable in many respects. His "Yes" and his "No," his "Probably" and his "Perhaps," are the fine gold of language. He always makes every effort of which his mind is capable to arrive at correct and complete knowledge: and whatever be the condition of his knowledge he uses his utmost endeavours in the choice of words, phrases, and illustrations to xpress that condition with the strictest exactness. o judge of any court is more painstakingly impartial n his summing up of evidence, and no judge has the me assurance of the value of the evidence. It may e said generally that the typical scientific man, on his wn subject, is practically infallible: that is to say, at his pronouncements on that subject may be taken as thoroughly reliable, with due limitation for the state of knowledge.

Such a scientific man it is the aim of the modern physician to be. The medical knowledge of recent times is *knowledge*, not hypothesis, theory, probability, and guesswork. It is true that hypothesis and probability are still too often the chief guides a medical man has in the practice of his art; but then he never mistakes these for knowledge. He makes the clearest possible distinction in his own mind between assured knowledge and plausible hypothesis. When he has certainties for his guides his step is confident, his action assured. When probability is his only beacon-light he is cautious, tentative, and critically expectant. The ancient physicians were not always of this scientific temper.

With the increased and increasing popularity of science there has sprung up a growing curiosity about medicine, both as an exact science and as a practical rt. Intelligent people who have some familiarity vith the works of Darwin, Spencer, and other vriters of the same school, acquire first a semi-cientific taste for such studies, and then a direct ersonal interest in them as bearing upon them-lves. The science of human physiology in its application to the art of medicine has many things of great importance to say on questions of surpassing personal interest. The hereditary transmission of mental and physical peculiarities; the capacity for intellectual and manual work; the art of healthy living; the expectation of life; moral responsibility as influenced by the animal organism —these and allied questions are now in every educated person's mouth, and the universal discussion of them produces a keen and eager desire in many minds for a deeper and truer understanding of their general significance, and for a greater and more varied power of putting them to every variety of experimental test.

What is the use of increased knowledge of man, his structure, his origin, and his destiny? Will it make life less arduous, less full of risks, more hopeful, more successful? Whither does such science tend? Does it reach out to strength, courage, and victory in the strife; or does it simply gratify a morbid curiosity, and leave as a practical result an enfeebling weariness of the flesh and a gloom of the spirit more paralysing and hopeless still?

Medical science can answer many of these questions, and she is an oracle who knows nothing of vague prophecies and cautious ambiguities. It is true that there is in the ranks of medicine much of what Huxley has recently called "pseudo-science." Practitioners of the medical art, both licensed and unlicensed, lay hold of some newly discovered fact or temporary hypothesis, and with brazen cunning use it as the birdcatcher uses his innocent decoy, for the purpose of filling their own worthless palms with gold. Such persons often bring both science and practice into unmerited contempt. The non-scientific and the half-scientific public would be thankful for some kind of scientific Greenwich time by which they could set their own watches at convenient periods. They are constantly wishing to know the exact time of day according to the authoritatively corrected horologe, but they do not know where to look.

So far as the science and art of medicine are concerned, the general hospitals may be regarded as the town-clocks. Unfortunately they are clocks without dial-plates and hands, or at any rate without such as the public can see. The more enlightened members of the medical profession, both in general and special practice, do manage by dint of great and constant peering, and the use of a variety of field and other glasses, to keep themselves informed of the time of day; but the great body of practitioners, and still more the public, are generally from a few days to a hundred years behindhand.

There is much need of a general illuminated dial-plate and of two distinct hands. Such a dial-plate and hands The Hospital will endeavour to be. There are other well-known medical papers which are constantly giving descriptions of the inner mechanism of the clocks, and by implication making it more or less clear that the hands would be pointing —if there were any—in such and such a direction. That is a worthy and excellent function to perform, and we do not propose to interfere with it in any way. Our function will be, from time to time, to make known to all intelligent persons, both medical and lay, the march of time in the medical world, and in the scientific world in so far as it is related to the art and practice of medicine. We shall make no attempt to take the line of teaching every man to be his own doctor. The man who is his own doctor has generally a fool for his patient. But it is abundantly evident, and as satisfactory as it is evident, that there is a general desire to draw aside the veil of mystery behind which the art of medicine has too often been carried on, and to let the public see that now at any rate the days of conjuring and occult practices have come to an end. Modern medicine is full of candour and simplicity, and says to all men, "I can do much to keep you well, I can do much to make you well: but I cannot do all. You have your part to perform: and this you can perform all the better if you will open your eyes and understand what I am trying to do for you."

Physic, it must be admitted, is a hard study; that is, in its largest sense, which includes anatomy, physiology, pathology, diagnosis, and treatment. The true physician knows nothing of "finishing schools." That man who ceases to be a student when he has received the "tin case" containing his diploma from the registrar, must be absolutely hopeless. No man, by four, five, or six years' hospital study can be anything more than a mere beginner. Whatever be his degree, and however many his medals and honours, he knows only the rudiments of his art. He has laid the foundation and nothing more. There are some few persons who think that because they have got what they consider the highest, or one of the highest qualifications, therefore they are already men of high capability and dignity in the profession. They have yet to learn the very elements of human experience. The academy is not the world: study is not practice. Just as practice without study is dreary, so reading without enthusiasm is dull and stale. If The Hospital can rouse, stimulate, and incite to fresh enthusiasm those who by hard toil or distance from intellectual centres are in danger of falling behind in the race, it will perform no mean function in the economy of science and medicine.

